# Physiological and pathological functions of TMEM106B in neurodegenerative diseases

**DOI:** 10.1007/s00018-024-05241-z

**Published:** 2024-05-06

**Authors:** Min Zhu, Guoxin Zhang, Lanxia Meng, Tingting Xiao, Xin Fang, Zhentao Zhang

**Affiliations:** 1 Department of Neurology, Renmin Hospital of Wuhan University, Wuhan, 430060 China; 2 Department of Neurology, the First Affiliated Hospital of Nanchang University, Nanchang, 330000 China; 3 TaiKang Center for Life and Medical Sciences, Wuhan University, Wuhan, 430000 China

**Keywords:** Transmembrane protein 106B, Neurodegenerative diseases, Lysosome, Amyloid fibrils, Neurotherapy

## Abstract

As an integral lysosomal transmembrane protein, transmembrane protein 106B (TMEM106B) regulates several aspects of lysosomal function and is associated with neurodegenerative diseases. The TMEM106B gene mutations lead to lysosomal dysfunction and accelerate the pathological progression of Neurodegenerative diseases. Yet, the precise mechanism of TMEM106B in Neurodegenerative diseases remains unclear. Recently, different research teams discovered that TMEM106B is an amyloid protein and the C-terminal domain of TMEM106B forms amyloid fibrils in various Neurodegenerative diseases and normally elderly individuals. In this review, we discussed the physiological functions of TMEM106B. We also included TMEM106B gene mutations that cause neurodegenerative diseases. Finally, we summarized the identification and cryo-electronic microscopic structure of TMEM106B fibrils, and discussed the promising therapeutic strategies aimed at TMEM106B fibrils and the future directions for TMEM106B research in neurodegenerative diseases.

## Introduction

Transmembrane protein 106B (TMEM106B), composed of 274 amino acids, is a type II transmembrane lysosomal protein with its subcellular location being in the late endosome and lysosomal membranes [1, 2]. It is found primarily within neurons and oligodendrocytes in the central nervous system [3]. It has been reported that TMEM106B is an integral lysosomal protein and has crucial effects on lysosome morphology, localization, trafficking, and functions [2, 4, 5]. Although its exact role in the pathogenesis of neurodegenerative diseases remains unclear, studies have shown that TMEM106B appears to affect the pathological burden of TAR DNA binding protein-43 (TDP-43) pathology [5]. Previous studies found that the expression of TMEM106B (including mRNA and protein) in the brain of Alzheimer’s Disease (AD) patients is significantly reduced [6]. However, mutations in TMEM106B increase its expression level, result in lysosome dysfunction, and promote its aggregation [2, 4]. Genome-wide association studies (GWAS) identified mutations of the TMEM106B gene as a major risk factor for frontotemporal lobar degeneration with TDP-43 pathology (FTLD-TDP) [7, 8], which was mostly associated with the risk of FTLD-TDP in patients with progranulin (GRN) mutations [7, 9, 10]. Mutations and single nucleotide polymorphisms (SNPs) of the TMEM106B gene lead to lysosomal deficits in the clearance of misfolded proteins, which are the main pathological changes in multiple Neurodegenerative diseases [11–13]. Recently, studies revealed that the luminal domain of TMEM106B forms amyloid fibrils in various Neurodegenerative diseases and neurologically normal older adults [14]. However, the precise mechanism of TMEM106B at the lysosomal membrane is undetermined and it remains unknown how TMEM106B contributes to the development of Neurodegenerative diseases. In this review, we will introduce current knowledge of TMEM106B in physiological and pathological function and its potential association with Neurodegenerative diseases. Then, we elucidate the identification and cryo-electronic microscopic (cryo-EM) structure of TMEM106B fibrils and analyze the factors that contribute to the polymorphisms of TMEM106B fibrils. Finally, we discussed the potential pathogenic role of TMEM106B fibrils and the future directions for TMEM106B research in Neurodegenerative diseases.

## Structure of TMEM106B in the native state

**Fig. 1:**
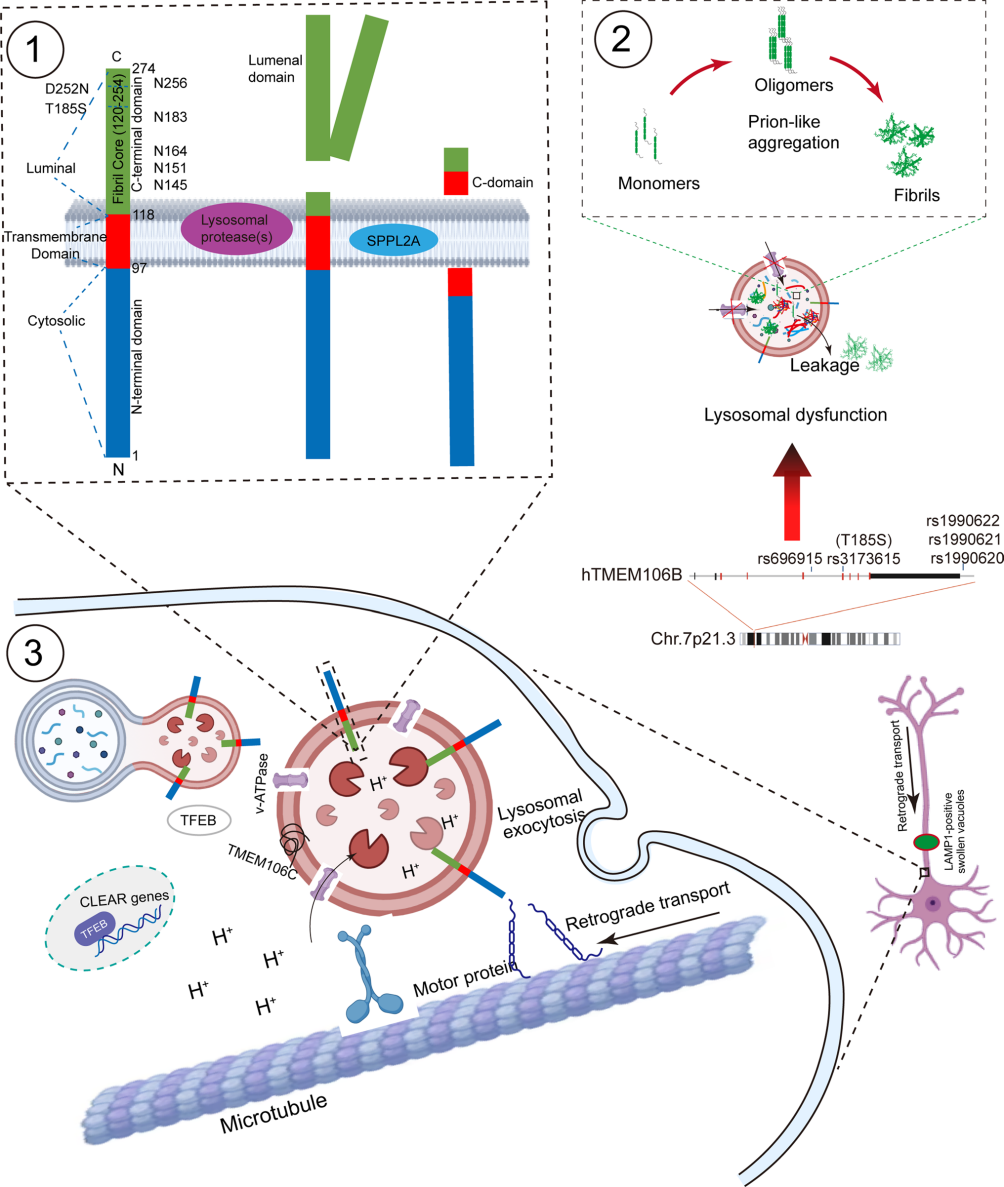
Structure, physiological and pathologic function of TMEM106B. ① TMEM106B is a type II transmembrane protein located on late endosome and lysosome, with its N-terminus facing the cytosol and C-terminus facing the lysosome lumen. TMEM106B consists of 274 residues and three structural domains. This C-terminal fragment (118–274 residue) contains five important N-glycosylation sites (N145, N151, N164, N183, N256). TMEM106B is processed into a N-terminal fragment (NTF) by lysosomal proteases, which is further cleaved by SPPL2a through intramembrane proteolysis to generate intracellular cytosolic domain. ② TMEM106B SNPs and mutations lead to TMEM106B abnormal expression and dysfunction in morphology, transportation, acidification and maturation of lysosomes. TMEM106B fragment may form fibrils after cleavage. ③Lysosomal activity is modulated by intraluminal pH. The interaction between TMEM106B and v-ATPase controls the acidification of lysosomes. TMEM106B knockdown results in less efficient fusion with autophagosome, poor protein degradation efficiency, and insufficient acidification. TMEM106B also regulates lysosomal trafficking in neurons. In dendrites, TMEM106B interacts with MAP6 to restrict retrograde transport of lysosomes. Loss of TMEM106B leads to the formation of LAMP1-positive lysosomal vacuoles at the axon initial segment region. This is partly due to increased retrograde transport of lysosomes along axons. TMEM106B further activates TFEB-dependent lysosome biogenesis. Endosome and lysosome fusion are also mediated by TMEM106B

TMEM106B is mainly expressed on the lysosomal membranes of neurons and oligodendrocytes in the central nervous system [15]. This protein consists of three parts, an N-terminal domain (NTD, 1–96 aa) facing the cytosol, a transmembrane domain (TMD, 97-117aa), and a C-terminal domain (CTD, 118–274 aa) facing the lysosome lumen [1]. TMEM106B is likely to be processed by lysosomal proteases at a position close to G127 [2]. The resulting C-terminal fragment contains five highly glycosylated sites at N145, N151, N164, N183 and N256 [1]. Following shedding of the ectodomain, the residual N-terminal fragment (NTF) is anchored to the lysosomal membrane, which is further cleaved by signal peptide peptidase-like 2A (SPPL2A) through intramembrane proteolysis to release intracellular cytosolic domain [16] (Fig. 1). However, many questions remain on (i) which protease(s) is responsible for cleaving the luminal domain, (ii) the precise cleavage site, (iii) the factors that contribute to enzyme digestion of TMEM106B, and (iv) the potential functions of the generated peptides. The mechanisms behind the proteolysis of TMEM106B are important because this contributes to the understanding of what gives rise to TMEM106B fibrils formation.

Recent studies found that TMEM106B is made up of a single rod-like structure or a doublet of filaments forming a twisted ribbon, of which several polymorphisms have been identified: 4 singlets and 2 doublets [12]. The structural domains are thought to be related but have little to do with the N-terminal domain, as the TMEM106B gene polymorphisms are conserved at the N-terminus domain, and the differences in structure are primarily within the C-terminal region [12]. Misfolding and aggregation of ND-related proteins (amyloid-β, phosphorylated tau, α-synuclein, and TDP-43) result in the formation of filamentous cellular inclusions. These abnormal amyloid fibrils are resistant to sarkosyl and found to be pathogenic hallmarks of Neurodegenerative diseases. A better observation of the molecular structures of these pathogenetic proteins strengthens our understanding of neuropathogenesis. Recently, the cryo-EM results revealed that TMEM106B protein belongs to amyloid proteins and has the potential to form amyloid fibrils. A new amyloid fibril composed of a C-terminal fragment of TMEM106B was found in many Neurodegenerative diseases and normal elderly [17–20]. Previous studies found that the TDP-43 protein is the pathological protein in FTLD-TDP disease, but recent studies indicate that the disease is associated with amyloid fibrils that are formed by TMEM106B [19]. This makes the TMEM106B protein a recent research hotspot.

## TMEM106B modulates the physiological function of lysosomes

The lysosome is a degradative organelle that acts as the waste disposal system by clearing out debris and unnecessary proteins. A typical signature of TMEM106B is its post-translational glycosylation. TMEM106B contains five putative consensus sequence motifs for N-glycosylation and additional modifications that are correlated with the location of lysosome [1]. The effects of glycosylation at different sites of TMEM106B are diverse. Glycosylation is partially required for the transport of TMEM106B beyond the endoplasmic reticulum to late cellular compartments. Mutations of the noncomplex glycosylation sites (N145, N151 and N164) do not affect the localization of TMEM106B. However, glycosylation of residues N183 and N256 may regulate its lysosomal localization. Moreover, glycosylation at N183 appears to be required for the anterograde trafficking and guides the normal transport of TMEM106B to late endosomes/lysosomes, the deficiency of which leads to the accumulation of TMEM106B in the endoplasmic reticulum [1, 21, 22]. These data indicate that N183 glycosylation is responsible for the anterograde transport of TMEM106B to late endosomes/lysosomes. N256 glycosylation causes TMEM106B localization to the cell surface, suggesting a direct effect of N256 glycosylation on the sorting of TMEM106B into endosomes [1].

Ectopic expression of TMEM106B induces morphologic changes in lysosome compartments and delays the degradation of endocytic cargoes by the endolysosomal pathway [2]. TMEM106B deficiency reduces the number of lysosomes, and the remaining lysosomes cluster at the axon initial segment or perinuclear space with a vacuole-like morphology [22, 23]. Low-expression of TMEM106B results in a disruption of lysosome maturation, presented as less efficient fusion with autophagosome, poor protein degradation efficiency, and insufficient acidification [24]. Intriguingly, cells over-expressing TMEM106B result in the loss of vacuolar phenotype and exhibit impaired lysosomal acidification and degradative function [21]. Furthermore, TMEM106B overexpression enhances oxidative stress-induced cytotoxicity [11, 25, 26], induces lysosomal enlargement, and results in cell death [1, 2, 27]. Neuronal TMEM106B overexpression enlarges LAMP1-positive structures and alters lysosomal stress signaling, causing a translocation of transcription factor EB to neuronal nuclei and increasing the expression of Coordinated Lysosomal Expression and Regulation (CLEAR) genes [22]. Similarly, TMEM106B moves from cytosolic and lysosomal compartments into the nucleus to activate lysosomal genes [28]. Reduction of TMEM106B increases axonal transported lysosomes, while TMEM106B elevation inhibits neuronal lysosomal transport, redistributes from the cell periphery to the perinuclear region, and yields large lysosomes in the soma [22].

Overexpression of TMEM106B results in abnormalities in endosomal-lysosomal dysfunction, such as endosome-lysosome morphology, acidification, and trafficking [29]. Since lysosome morphology and size are intricately regulated by fission events and fusion with other organelles [30], and may be partially dependent on pH and successful trafficking. Disorders of either fission or fusion events lead to clustering lysosomes and the formation of large swollen vacuoles [13]. This indicates that TMEM106B may regulate many aspects of lysosomal function, including lysosomal pH, lysosome movement, and lysosome exocytosis [11]. Usually, lysosomes are transported along microtubules with the help of the motor proteins. TMEM106B was demonstrated to interact with microtubule-associated protein 6 (MAP6). This may inhibit the retrograde transport of lysosomes, facilitate appropriate transport of lysosomes, and prevent transport along microtubules through motor proteins [31]. By analyzing the interaction between TMEM106B and MAP6, researchers found that overexpression of TMEM106B affects bidirectional transport, in particular favoring retrograde transport [2]. Furthermore, the lysosome maintains its acidic environment by pumping in protons (H^+^ ions) from the cytosol via the vacuolar-ATPases (vATPase). TMEM106B overexpression interacts with accessory proteins of vATPase, resulting in a reduction in vATPase activity and the destruction of an acidic environment in lysosomes [11]. Interestingly, the inhibition of vATPases significantly increases the levels of TMEM106B [1]. A diagram is presented to illustrate the physiological and pathological roles of TMEM106B on lysosomes (Fig. 1).

## Multiple single nucleotide polymorphisms (SNPs) of TMEM106B

Aberrant TMEM106B expression and deposition were detected in neurodegenerative diseases. GWAS showed that variants of the human TMEM106B gene are risk factors for FTLD-TDP, especially in patients with granulin (GRN) mutation [7]. Six SNPs of TMEM106B were found to modify the disease risk for several Neurodegenerative diseases and are associated with their clinical and pathological phenotypes [32–36]. Five out of these SNPs are located in the non-coding regions of TMEM106B and regulate its expression by influencing the alternative splicing of TMEM106B mRNA. Experimental evidence suggests that variants on TMEM106B haplotypes increase its expression, and are strongly correlated with pathological phenotypes and disease risk of FTLD-TDP. Genotyping data revealed that the risk allele of rs1990622, located in the 3'-untranslated region of TMEM106B, was a risk factor for FTLD-TDP, and carriers of rs1990622 risk allele increase TMEM106B protein levels in the hippocampus with physiological aging [37]. The T allele at this position is considered the major isoform, linked to higher risks of developing Neurodegenerative diseases or exacerbated cognitive decline. In contrast, the minor C allele is associated with a protective phenotype [9, 10, 34]. The levels of TMEM106B mRNA and protein were significantly increased in GRN mutation carriers [27, 38]. The major allele of rs1990622 in TMEM106B is associated with later age of onset and death of FTLD patients with C9orf72 mutation [32]. Still, this effect was not observed in FTLD-TDP patients without GRN or C9orf72 mutation, indicating different roles of TMEM106B in FTLD. Neuroimaging studies have shown reduced left hemispheric grey matter volume in those carrying the A allele of rs1990622 within a healthy population cohort [39], but it is still unclear whether it affects the volume of key brain areas in AD patients. Genotyping data revealed that the frequency of homozygous minor alleles was significantly reduced in GRN mutation carriers [40]. This result was confirmed in the C9orf72 expansion carriers [36, 41, 42]. In amyotrophic lateral sclerosis (ALS), the minor allele of rs1990622 shows more severe cognitive impairments, and poor motor functional status [43–45]. However, another study found the contrary results that ALS patients with the major allele of rs1990622 showed better cognition but worse motor functions than patients homozygous for the minor allele [46]. Research conclusions on the effect of TMEM106B SNPs on AD are inconsistent. Yang and colleagues conducted an expression quantitative trait loci (eQTLs) analysis of rs1990622 variant and found that rs1990622 variant T allele could increase TMEM106B expression [47]. However, Satoh et al. demonstrated both the mRNA and protein levels of TMEM106B were significantly reduced in AD brains compared to controls [48, 49]. Hu and colleagues analyzed the GWAS datasets and reported rs1990622 variant T allele only contributes to increased AD risk in females, but not in males. In addition, rs1990622 variant could regulate the expression of TMEM106B in human brain tissues, which vary considerably in different disease status [35]. Moreover, case–control association studies found that the risk allele of rs1990622 confers increased susceptibility to late-onset AD in the apolipoprotein E (APOE) ε4 allele carriers [50]. Furthermore, GWAS analyses identified a novel genome-wide significant association with genetic markers in DNA variants of TMEM106B and neurofilament light chain (NfL) protein concentrations in the cerebrospinal fluid of AD patients [51], but the relationship between TMEM106B and Aβ/tau proteins in cerebrospinal fluid (CSF) of AD patients remains to be explored. Intriguingly, recent two in vivo studies investigated the role of TMEM106B in tau P301S transgenic mouse model. Feng et al. show that loss of TMEM106B enhances the accumulation of pathological tau and results in severe neuronal loss, especially in the neuronal soma in the hippocampus [52]. The other found similar results that TMEM106B deletion accelerates cognitive decline and tau pathology in tau P301S mice. In contrast, the T186S (equivalent to the human T185S variant) knock-in mutation protected against tau-associated cognitive decline and synaptic impairment [53]. In PD, the rs1990622 risk allele was linked to a greater and faster cognitive deterioration [44]. The variant rs1990622 was also reported to correlate with reduced neuronal degeneration during aging, independently of disease [54].

Moreover, a noncoding variant, rs1990620, was shown to preferentially recruit the chromatin-organizing protein CCTC-binding factor (CTCF) to modulate TMEM106B expression through transcriptional activation due to CTCF-mediated long-range chromatin-looping interactions [25]. It was also shown that one coding variant rs3173615 encoding a threonine to serine change at amino acid position 185 (p.T185S) contributes to the disease-modifying effect [34, 41]. The change of T185 to serine was found to protect against FTLD-TDP [55], possibly because the protein with a serine is more rapidly degraded [56].TMEM106B carrying the risk isoform T185 leads to a higher level of glycosylation at N183, affecting the protein stability and degradation [10, 56]. Alternatively, the coding p.T185S variant might influence disease risk by altering either TMEM106B biology (cleavage, dimerization, etc.) or by affecting lysosomal dysfunction. The T185 variant seems to enhance the binding ability of S185 variant to the charged multivesicular body protein 2B (CHMP2B), which led to a decrease in autophagic flux [57]. Furthermore, overexpression of TMEM106B T185 delayed the degradation of the endogenous epidermal growth factor receptor, suggesting that TMEM106B may drive a defect in late endosome/lysosome fusion or lysosomal degradation [2]. A study revealed that AD risk is significantly influenced by the interaction of APOE with rs1595014 in TMEM106B [58]. The rs1990621, another SNP of TMEM106B, was identified as a protective variant against general aging, independent of disease status [54]. In chronic traumatic encephalopathy (CTE) patients, the homozygous carriers of the major allele of rs3173615 in TMEM106B appear to be more severe in tau pathology than those with the minor allele [59]. This finding suggests that TMEM106B variants modify tau pathology in CTE patients. However, another study reported a negative role of the genetic variations of rs3173615 in TMEM106B in CTE patients, compared to controls [60]. TMEM106B knockdown restores endolysosomal trafficking and branching defects [61]. Furthermore, a novel dominant D252N mutation in TMEM106B causing an amino acid substitution has been recently associated with several cases of hypomyelinating leukodystrophy (HLD) [62, 63]. The SNPs D252N was shown to abolish lysosome enlargement and lysosome acidification induced by wild-type TMEM106B overexpression in oligodendrocytes [15], suggesting that D252N mutation may impair lysosomal function by altering the autophagy process. In addition to chromatin structure alterations, the microRNA-132/miRNA-212 cluster binds to the 3′UTR of TMEM106B gene and inhibits its expression [27]. The microRNA is significantly decreased in FTLD-TDP, thus suggesting an upregulation of TMEM106B expression [64]. Table 1 summarizes the Cohort studies about TMEM106B SNPs and its potential phenotypes.

**Table 1:** Summary of Cohort studies about TMEM106B SNPs and its potential phenotypes

Disease		SNPs/mutations		major allele		minor allele		Phenotypes associated with SNPs/mutations	Cohort composition	
FTLD-*GRN*	rs1990622		A		G		Increased onset of FTLD-GRN at an earlier age [55].			FTLD-GRN + : *n* = 27; FTLD-GRN-: *n* = 23; Controls: *n* = 73
	rs1990622		T		C		Reduced the disease penetrance in patients with GRN mutations [10].			FTLD-GRN + : *n* = 78; FTLD-GRN-: n = 562; Controls: *n* = 822
	rs1990622		A		G		Decreased cortical volumes in FTLD-GRN patients [33].			FTLD: *n* = 198
	rs1990622		T		C		Associated with decreased connectivity within the ventral salience network and the left frontoparietal network [40].			GRN + : *n* = 17; Controls: *n* = 14
	rs1990622		A		G		Protective role of TMEM106B in GRN carriers [65].			GRN + : *n* = 76; FTLD: *n* = 384 Controls: *n* = 552
	rs1990622		T		C		Associated with Worse cognitive decline in FTD and PD, but not in AD and MCI. [44].			PD: *n* = 179; FTD: *n* = 179; AD: *n* = 300; MCI: *n* = 75; Controls: *n* = 137
	rs1990622		T		C		Conferred risk of FTLD-TDP by increasing TMEM106B expression [7].			FTLD-TDP: *n* = 515; Controls: *n* = 2509
	rs1990622		T		C		Homozygous carriers of the Minor allele reduced the risk of developing FTLD-TDP [9].			FTLD: *n* = 288; Controls: *n* = 595
	rs1990621		C		G		Associated with increased neuronal proportion [54].			Discovery cohort: AD: *n* = 317; Controls: *n* = 100. Replicate cohort: AD: *n* = 323; FTLD: *n* = 11; Controls: *n* = 388
	rs3173615 (T185S)		C		G		The protective haplotype has about 50% lower odds of developing disease symptoms [34].			Discovery cohort: GRN + : *n* = 382; Controls: *n* = 1146; Replicate cohort: GRN + : *n* = 210; Controls: *n* = 1798
FTLD-* C9orf72*	rs1990622		T		C		Major allele associated with later onset and age at death [32].			FTLD-TDP: *n* = 241; C9orf72 + : *n* = 75
	rs3173615		G		C		Homozygosity of the minor allele protects C9orf72 expansion carriers from developing FTLD, but not from developing motor neuron disease [41].			Cohort 1: FTLD-C9orf72 + : *n* = 325; Cohort 2: FTLD-C9orf72 + : *n* = 586; Control: *n* = 1302
	rs1990622		A		G		No association between TMEM106B genotypes and C9orf72 repeat expansions [65].			C9orf72 + : *n* = 145; Controls: *n* = 552
ALS	rs1990622		T		C		Associated with declined cognitive function [45].			ALS: *n* = 85; Controls: *n* = 553
	rs1990622		A		G		The minor allele had less bulbar site of onset, but had severe cognitive impairment [46].			ALS: *n* = 865
	rs1990622		T		C		The minor allele homozygotes had more TDP-43 pathology [36].			ALS: *n* = 110
AD	rs1990622		T		C		The minor allele had a low frequency in AD cases with TDP-43 pathology [66].			AD: *n* = 907
	rs1990622		T		C		Associated with LOAD in the APOE ε4 carriers [50].			LOAD: *n* = 1133; Controls: *n* = 1159
	rs1990620		A		G		Reduced inflammation in LOAD [67].			LOAD: *n* = 419; Controls: *n* = 849
	rs1990622		T		C		The minor allele was less often in HpScl and HpScl-AD than the typical AD and LP-AD [68].			Typical AD: *n* = 807; LP-AD: *n* = 151; HpScl-AD: *n* = 132; HpScl: *n* = 30
	rs1990622		T		C		Major allele contributed to increased AD risk in females, but not in males [35].			AD: *n* = 21,982; Controls: *n* = 41,944
	rs1595014		A		T		AD risk is significantly influenced by the interaction of APOE with rs1595014 in TMEM106B [58].			Discovery cohort: AD: *n* = 17,536; Controls: *n* = 36,175; Replicate cohort: AD: *n* = 13,219; Controls: *n* = 4116
	rs1548884		C		A		Associated with CSF NfL levels [51].			Discovery cohort: AD: *n* = 154; MCI: *n* = 401; Controls: *n* = 122; Replicate cohort: AD: *n* = 70; MCI: *n* = 151; Controls: *n* = 87
	rs1990622		A		G		Associated with AD pathology [69].			Aging without FTLD: *n* = 544
PD	rs1990622		T		C		Associated with worse cognitive decline in PD [44].			PD: *n* = 179; Controls: *n* = 137
	rs1990622/rs3173615		C		T		Associated with initial symptoms of rigidity/bradykinesia in PD patients [70].			PD: *n* = 1121; Controls: *n* = 829
HS-aging	rs1990622		A		G		Homozygosity for the major allele genotypes carriers have a higher risk for developing HS-Aging pathology [71].			HS-Aging: *n* = 268; Controls: *n* = 2957
	rs1990622		A		G		Increased risk and more advanced TDP-43 pathology [69].			Aging without FTLD: *n* = 544
CTE	rs3173615		C		G		Less inflammation and ante-mortem dementia in CTE patients [60].			CTE: *n* = 86
	rs3173615		C		G		Homozygous carriers of minor allele had less CTE pathology [59].			CTE: *n* = 66; Controls: *n* = 198
LATE	rs1990622		T		C		Associated with severe loss of CA1 neurons [72].			LATE-NC: n = 1457
	rs1990622		T		C		Increased risk to LATE-NC [73].			Community-based autopsy cohorts: *n* = 1309
Aging	rs1990622		A		G		One of major dementia risk genes that affects glial lipid metabolism [74].			Neurologically normal human aging: *n* = 74

*AD* Alzheimer's disease, *ALS* amyotrophic lateral sclerosis, *CSF* cerebrospinal fluid, *CTE* chronic traumatic encephalopathy, *FTLD* frontotemporal dementia, *FTLD-TDP* frontotemporal lobar degeneration with TAR DNA-binding protein inclusions, *GRN* granulin, *HpScl* hippocampal sclerosis, *HS-Aging* hippocampal sclerosis of aging pathology, *LOAD* late-onset Alzheimer disease, *LATE* limbic-predominant age-related TDP-43 encephalopathy, *LP-AD* limbic-predominant Alzheimer disease, *MCI* mild cognitive impairment, *PD* Parkinson disease, *TDP-43* TAR DNA-binding protein 43

## The cryo-EM structure of TMEM106B fibrils

In the past two years, several research groups have reported that TMEM106B forms amyloid fibrils in the brain tissue of different neurodegenerative diseases and elderly normal subjects through cryo-EM [17–20]. These observations indicate that amyloid fibrils formed by TMEM106B may play a role in the pathogenesis of neurodegenerative diseases. Interestingly, the methods used for the identification of TMEM106B vary among different groups. Chang et al. supplemented cryo-EM with mass spectrometry to identify TMEM106B peptides present in Sarkosyl-insoluble components of FTLD-TDP, progressive supranuclear palsy, and dementia with Lewy bodies [17]. Jiang et al. modeled two query sequences and determined their structures based on cryo-EM density in FTLD-TDP subclasses [19]. Fan et al. found TMEM106B forms amyloid fibrils not only in diseased brains, but also in the brains of normal elders by cryo-EM [18]. Significantly, the burden of TMEM106B fibrillization is much higher in most individuals with Neurodegenerative diseases, when compared with age-matched healthy controls. These data indicate that the TMEM106B fibrils may not be a by-stand in neurodegeneration, but might exert some toxic effects, and contribute to age-dependent neurodegeneration. Schweighauser and colleagues detected TMEM106B filaments based on the unique glycosylation pattern of fibrils [20]. However, all reports observed that amyloid fibrils have ordered cores containing TMEM106B residues S120-G254. These studies suggest that the cleavage between residues 119 and 120 is essential for fibril formation, because the cleaved terminus is buried in the fibril core, leaving no space for other residues. All papers showed that the TMEM106B fibrils stack into protofilaments-single protofilaments form rod-like structures, and pairs of protofilaments form twisted ribbons. The interface in the more prevalent paired fibrils was mediated by residues K178 and R180 of each protofilament interacting with an unresolved density, potentially an anionic cofactor.

Although the TMEM106B fibrils are extracted from the insoluble portion of sarcosine in postmortem tissue, there are some differences in fractionation schemes, including the sarcosine addition stage, the use of ultracentrifugation or low-speed centrifugation, and changes in heating or streptomycin treatment. Jiang et al. extracted and determined the amyloid fibrils from brains of FTLD-TDP patients by cryo-EM [19]. Unexpectedly, all amyloid fibrils were composed of a 135-residue carboxy-terminal fragment of TMEM106B, but not TDP-43 filaments. Jiang et al. used immunogold labeling and identified abundant non-fibrillar aggregated TDP-43 in FTLD-TDP patients [19]. Considering the discrepancy in extraction methods, TDP-43 filaments may be lost during the extraction process and were not obtained in the analyzed process [75]. This emphasizes the importance of sample preparation schemes in cryo-EM research and indicates the need for more samples aiming at analyzing TDP-43 aggregates. Also, different methods should be conducted to verify the amyloid protein properties of TMEM106B in various Neurodegenerative diseases.

## Future direction on TMEM106B in neurodegenerative diseases

Several studies identified that TMEM106B may play a role in the pathogenesis of neurodegenerative diseases. However, the exact molecular mechanisms remain unknown. Whether the presence of these fibrils correspond solely to age regardless of whether or not a person has neurodegenerative conditions, or is specifically related to a specific disease state, remains unknown. None of the current studies involves enough autopsy samples to have a statistical power to associate TMEM106B fibrils with FTLD-TDP. TMEM106B fibrils are deposited in many brain regions of patients of various Neurodegenerative diseases, but the mechanism by which TMEM106B fibrils contributes to neurodegenerative pathology is currently unclear. Further research is needed to determine whether there is regional or disease-specific susceptibility for TMEM106B aggregation, and how these aggregates evolve and spread with disease progression. It is possible that increased/reduced TMEM106B expression might affect lysosomal trafficking and neuronal development [76]. However, it remains unclear whether the deposition of TMEM106B fibrils directly leads to neurodegeneration, or does it promote the aggregation and deposition of other pathological proteins, such as Aβ, tau, and α-syn. TMEM106B fibrils are also deposited in neurologically normal older adults, so what is the role of TMEM106B in the elderly, an initiator or a bystander? These issues need to be discussed.

Given that TMEM106B plays an important role in the pathological progression of Neurodegenerative diseases, TMEM106B may be used as a therapeutic target. Finding factors that inhibit the initial aggregation of TMEM106B fibrils will be the focus of future research. The C-terminal fragment of TMEM106B form fibrils. Thus, reducing the generation of the C-terminus of TMEM106B would be an effective way to block the fibrilization process. SPPL2a is a key protease in cleaving TMEM106B, and its antagonist could be a target to block the production of C-terminal fragment [16]. Post-translational modifications could mediate the structural diversity of fibrils by influencing their inter-protofilament interfaces^78^. Glycosylation is partially required for the transport of TMEM106B beyond the endoplasmic reticulum to late cellular compartments. Complex glycosylation at the N183 site appears to affect anterograde trafficking, whereas N256 site glycosylation appears to influence the direct sorting of TMEM106B to endosomes [1]. Therefore, to regulate the post-translational glycosylation modification of TMEM106B may be a potential therapeutic approach. Third, looking for conformation-specific antibodies and potential small molecules to reduce the formation of TMEM106B β-sheet structure, which is the initial step in inducing amyloid aggregation, may prevent TMEM106B fibrillation. Lysosomes are responsible for the degradation of TMEM106B fibrils and lysosomal dysfunction may alter fibrillation by affecting pH and protease activity. Also, lysosomal dysfunction may result in an increase in intraluminal and lysosomal exocytosis of TMEM106B fibrils due to impaired degradation. Thus, promoting the degradation of TMEM106B fibrils through the lysosome pathway would be promising.

## Conclusions

The deposition of TMEM106B fibrils identified by Cryo-EM will undoubtedly highlight the role of TMEM106B in neurodegenerative diseases, but there are many questions to be answered. First, TMEM106B fibrils are present in older adults without neurodegenerative disease, and their pathogenicity needs to be further determined. Second, as TMEM106B fibrils have been found in various Neurodegenerative diseases, it needs to be clarified whether TMEM106B fibrils have synergistic or antagonistic effects on promoting aggregation of pathological proteins in Neurodegenerative diseases. Third, TMEM106B risk haplotype may affect fiber load by modulating TMEM106B expression or processing, or directly by affecting glycosylation at the N183 site. Detailed studies of haplotype contributions are needed. Finally, it would be necessary to elucidate the mechanism of TMEM106B C-terminus generation, the precise steps, the proteases, and cellular conditions required for TMEM106B fibrillization, and to determine the precise cell location of TMEM106B fibrils. Further work will be required to explain these ambiguities and determine whether the fibrils are harmful, irrelevant, or even protective against disease.

## Data Availability

Not applicable.
